# The linear artifact in enhanced depth imaging spectral domain optical coherence tomography

**DOI:** 10.1038/s41598-017-08811-3

**Published:** 2017-08-16

**Authors:** Chengguo Zuo, Lan Mi, Shasha Yang, Xinxing Guo, Hui Xiao, Xing Liu

**Affiliations:** 1 State Key Laboratory of Ophthalmology, Zhongshan Ophthalmic Center, Sun Yat-sen University, Guangzhou 510060, China; 2 Guangzhou First People’s Hospital, Guangzhou Medical University, Guangzhou, China

## Abstract

Optical coherence tomography (OCT) is a valuable ancillary test in the diagnosis and management of chorioretinal disease. The evaluation of choroid thickness using OCT has become the focus of clinical applications. We report a linear artifact that acts as a confounding factor in choroidal thickness measurements by enhanced depth imaging OCT. We found that the linear artifact is located stably at a depth of 485 *μ*m beneath the retinal pigment epithelium in 81.88% of subjects. The study suggested that the linear artifact was a confounding factor in assessing choroidal thickness and that caution should be used in the interpretation of the choroidal thickness, especially when it is approximately 485 *μ*m.

## Introduction

Optical coherence tomography (OCT) has become a valuable ancillary test in the diagnosis and management of chorioretinal disease^1^. It can be used to acquire high-resolution, cross-sectional, non-invasive and non-contact *in vivo* images and biometric indexes as well as quantify retinal thickness in different layers.

Spaide and colleagues developed the technique of enhanced depth imaging (EDI) OCT, which provides *in vivo*, high-resolution, optical and cross-sectional images of the choroid, including choroidal thickness and large choroidal vessels^2, 3^. The reliability and repeatability of the choroid images have been documented in eyes with and without ocular diseases and in patients of different races^4, 5^. Therefore, the EDI-OCT has been an emerging area of study in various ocular diseases involving the choroid, including central serous chorioretinopathy, acute primary angle closure, open-angle glaucoma, nanophthalmic eyes, etc.^6–9^.

Although the outer border of the retinal pigment epithelium (RPE) hyper-reflective band is clear for the measurement of the subfoveal choroidal thickness (SFCT), the sclerochoroidal interface is not always easy to distinguish^4^. In case of artifact, it is more difficult to determine the accurate choroidal thickness. We found a widespread artifact that easily blurred the sclerochoroidal interface in EDI-OCT in our clinical practice. But the artifact has never been reported before.

The purpose of our study was to report the location and possible cause of the linear artifact, which could be a confounding factor in choroidal thickness measurements.

## Results

### Demographic features of the participants

Among the 149 participants, 21 had acute primary angle closure glaucoma (APACG), 24 had primary angle closure suspect (PACS), 32 had chronic primary angle closure glaucoma (CPACG), 25 had primary open angle glaucoma (POAG), 25 had central serous chorioretinopathy (CSC) and 20 were control subjects (Table 1).

**Table 1:** Demographic features of study participants.

	APACG (n = 23)	PACS (n = 24)	CPACG (n = 32)	POAG (n = 25)	CSC (n = 25)	Control (n = 20)
Mean age, yrs	57.25 ± 12.60	56.22 ± 12.18	56.45 ± 9.24	55.08 ± 12.54	40.12 ± 6.57	64.20 ± 11.60
Male sex, n (%)	7 (30.43%)	7 (29.17%)	14 (60.87%)	9 (36%)	23 (92%)	16 (64%)
Diopter (D)	0.32 ± 1.58	0.47 ± 1.73	−0.41 ± 1.02	−1.08 ± 1.70	0.045 ± 1.25	−0.09 ± 0.93

The linear artifact was found in 122 cases (81.88%). All the detected linear artifact was located stably at a depth of 485 *μ*m beneath the RPE. The subfoveal choroidal thickness measured with EDI-OCT was 325.43 ± 140.98 *μ*m in these subjects (Table 2).

**Table 2:** Ophthalmologic data (mean ± SD) of study participants.

	APACG	PACS	CPACG	POAG	CSC	Control
Frequency of artifact, n (%)	12 (52%)	18 (75%)	23 (72%)	24 (96%)	25 (100%)	25 (100%)
Subfoveal choroidal thickness (μm)	319.39 ± 134.96	348.38 ± 145.15	336.00 ± 115.03	283.88 ± 91.36	495.00 ± 113.82	240.35 ± 106.32
Depth of the artifact (μm)	485	485	485	485	485	485
Height from ellipsoid zone to RPE/Bruch’s complex (μm)	79.83 ± 6.42	83.41 ± 6.04	82.17 ± 5.91	82.33 ± 5.14	77.00 ± 7.07	78.60 ± 4.54
Height of the artifact (μm)	61.50 ± 6.26	64.61 ± 5.56	64.13 ± 6.50	63.46 ± 4.85	62.00 ± 7.07	62.25 ± 4.71
Foveal retinal thickness (μm)	213.35 ± 14.90	213.25 ± 13.36	214.72 ± 13.26	217.68 ± 14.98	485.84 ± 151.15	208.85 ± 14.51

### Location of the linear artifact

**Figure 1:**
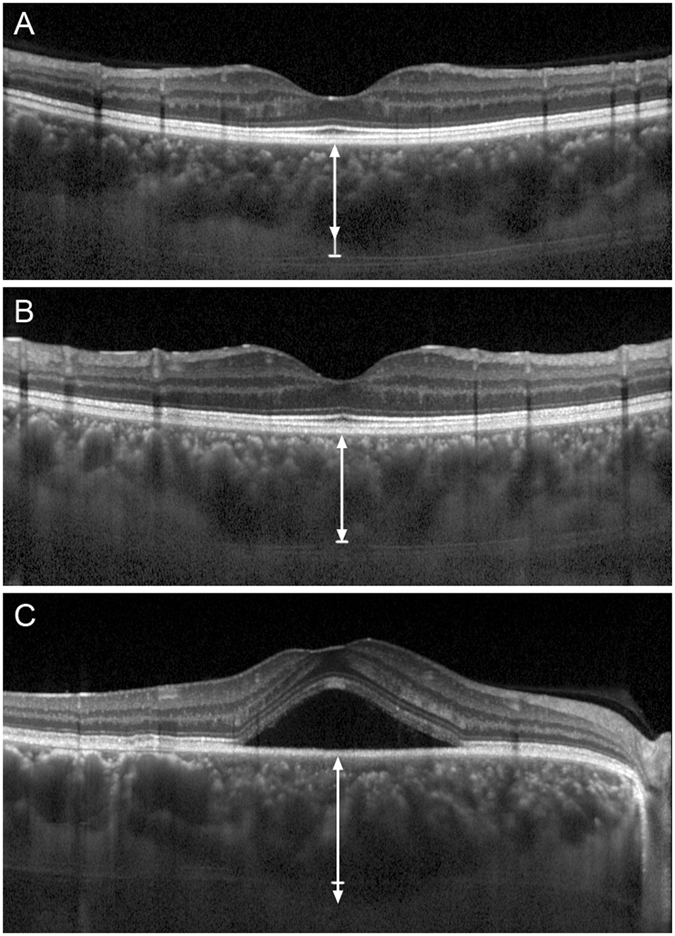
Example of a linear artifact line (small linear segment) below, at the same level as or above the sclerochoroidal interface (bidirectional arrow) in EDI-OCT images. (**A**) A female chronic primary angle closure glaucoma patient with a linear artifact line below the sclerochoroidal interface with a SFCT of 429 μm. (**B**) A control male subject in which the linear artifact was at the same level as the sclerochoroidal interface with a SFCT value of 485 μm. (**C**) A central serous chorioretinopathy patient with a linear artifact line above the sclerochoroidal interface with a SFCT of 573 μm. SFCT: subfoveal choroidal thickness.

The linear artifact could be observed below, at the same level as or above the sclerochoroidal interface, which was shown in Fig. 1.

**Figure 2:**
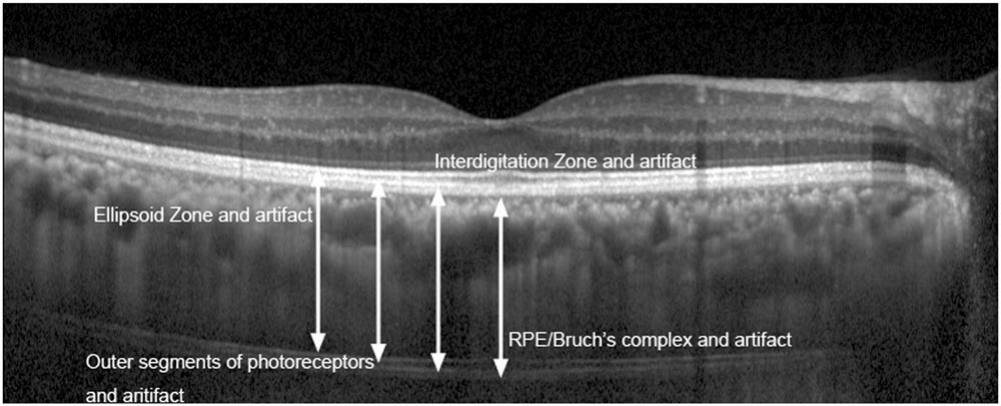
The linear artifact and corresponding retinal structures in EDI-OCT. The ellipsoid zone, the outer segments of photoreceptors, the interdigitation zone and RPE/Bruch’s complex corresponded to the four zones of linear artifact, respectively.

The distance between the inner and outer line of the linear artifact was 63.18 ± 5.52 *μ*m, and the height from the ellipsoid zone to RPE/Bruch’s complex was 81.30 ± 5.65 *μ*m. The linear artifact was like a miniature copy from the ellipsoid zone to the RPE/Bruch’s complex, and the reduction ratio was approximately 1:1.3 (Fig. 2). The rate of sclerochoroidal interface above, below or at the same level as the linear artifact line was 67.1%, 7.4% and 7.4%, respectively (Table 3).

**Table 3:** Rate of linear artifact line above, below or at the same level as the sclerochoroidal interface.

	APACG	PACS	CPACG	POAG	CSC	Control	Total
Below	10 (43.5%)	14 (58.3%)	19 (59.4%)	24 (96%)	14 (56%)	19 (95%)	100 (67.1%)
At the same level	1 (4.3%)	2 (8.3%)	3 (9.4%)	0 (%)	4 (16%)	1 (5%)	11 (7.4%)
Above	1 (4.3%)	2 (8.3%)	1 (3.1%)	0 (%)	7 (28%)	0 (0%)	11 (7.4%)
Not observed	11 (47.8%)	6 (25%)	9 (28.1%)	1 (4%)	0 (0%)	0 (0%)	27 (18.1%)

### Linear artifact in different SFCT groups

The participants were divided into two groups according to their SFCT. SFCTs less than 300 *μ*m were assigned to the normal SFCT group, while an SFCT equal to or greater than 300 *μ*m was assigned to the thickened SFCT group. The rate of sclerochoroidal interface below and at the same level as the linear artifact line was only 2.2% in participants in the normal SFCT group, such as POAG patients and control subjects, but the rate was upto 20.1% in patients in the thickened SFCT group, such as APACG, PACS, CPACG and CSC patients. The difference between the two groups was statistically significant (p < 0.01).

## Discussion

To date, EDI-OCT has become a promising new method in clinical practice^3^. The changes to the choroid support the hypothesis that hemodynamic changes in the choroid may cause many ocular diseases. A thicker choroid is observed in central serous chorioretinopathy, primary angle closure, nanophthalmic eyes and uveitis compared with that in normal eyes. A thinner choroid is found in APAC-afflicted eyes with elevated IOP compared with that in eyes with normal IOP^6, 7, 9–11^. Choroidal thickness therefore was thought to be meaningful in the assessment of disease severity and in predicting prognosis. Tagawa *et al*. observed a thickened choroid prior to the recurrence of Vogt-Koyanagi-Harada disease^11^. SFCT increase might be an anatomic feature of angle-closure disease^10^, but not of open angle glaucoma^12^. In longer and more myopic young adult eyes, the SFCT is thinner^13^ and may be a useful prognostic modality in high myopia^14^. The significantly increased SFCT was observed throughout the 6-month follow-up after cataract surgery^15^. Shao *et al*. even found an association between thin SFCT and subcapsular cataract or cortical cataract^16^.

There is still some disagreement about the results and corresponding interpretation of SFCT in many circumstances. SFCT was found to be negatively correlated with age, and the SFCT in males was 18% higher than in females in some investigations^17, 18^. However, a different study demonstrated no significant correlation between SFCT and age and sex, nor was there a significant correlation with ethnicity^19^. Since EDI-OCT is a new technique and increasing attention has been paid to choroidal thickness, the accurate determination of the boarder of the choroid is crucial. Accurate determination of SFCT is composed of two aspects: one is the outer boarder of the RPE, which is always clear and easy to define, and the other is the outer boarder of the choroid, which is not so clear and is easy to be confused by factors such as the linear artifact.

The occurrence frequency of the linear artifact in the present study was 81.88%. In some cases of APACG, the transparency of refractive media was affected by factors such as corneal edema and glaucomatous fleck, resulting in poor image quality. In these cases, the linear artifact could not be observed. In cases with relatively clear refractive media, such as PACS, CPACG and POAG, the rates were higher. In cases with clear refractive media, such as CSC and in the control group, the rate was 100%. The linear artifact can also be detected in many published literatures on EDI OCT, although it has never been reported^20–24^. This demonstrated that the incidence of the linear artifact was high and that the visibility of the linear artifact was affected by the opacity of refractive media.

In the present study, the linear artifact was stably located at 485 *μ*m from the outer border of the RPE hyper-reflective band in normal subjects and in subjects with many ocular diseases in which the SFCT is an important reference parameter. While the average SFCT in our study was 325.43 ± 140.98 *μ*m, the actual sclerochoroidal interface could be located above, below or at the same level as the linear artifact. Because the location of the linear artifact line was stable, the different relative positions mainly depended on differing SFCTs. When SFCT was thin, the sclerochoroidal interface was located above the linear artifact, and the artifact had little influence over the measurement of SFCT. However, when SFCT was thick, the sclerochoroidal interface was located at the same level of the linear artifact, and interfered with the measurement of SFCT. When SFCT was thicker, the sclerochoroidal interface was located below the linear artifact. Therefore, the artifact is most likely to be mistaken for the sclerochoroidal interface in the absence of careful observation or a clear image, especially in eyes with a thickened choroid, such as in APACG, PACS, CPACG and CSC. The rate of sclerochoroidal interface below and at the same level as the linear artifact line was upto 20.1% in patients with increased SFCT. The results of this study show that the linear artifact is a confounding factor in assessing the thickness of the choroid, especially in patients with increased SFCT.

In clinical practice, it is not always easy to distinguish the outer choroidal margin on obtained images. The combination of variations in patients, refractive transparency, image quality and anatomy can all affect the determination of the sclerochoroidal interface, which affects the determination of choroidal thickness. A repeatability study of manual SFCT measurements conducted by Rahman *et al*. observed that a change of more than 32 *μ*m was likely to exceed inter-observer variability in SFCT^4^. A previous investigation has shown relatively large SFCT measurement agreement in scans with a less visible choroidal outer boundary^5^. It illustrates the difficulty of locating the borderline of the choroid. To minimize the influence of the linear artifact, the following advice is recommended. First, capture images as clearly as possible. Second, evaluate images under the primary mode, that is, a white line on a black background. Finally, the average depth of the front interface of the linear artifact to the base of the RPE is 485 *μ*m, and the linear artifact is similar to an amplified copy of the ellipsoid zone, the interdigitation zone and the RPE/Bruch’s complex. We hypothesized that this artifact is derived from a mirror mapping of the bands from ellipsoid zone to RPE/Bruch’s complex because the reflexes of the bands from ellipsoid zone to RPE/Bruch’s complex are strong and they are imaged again after secondary reflection by the lens. Caution should be paid to the interpretation of the choroidal thickness, especially when the choroidal thickness is approximately or exceeds 485 *μ*m.

In conclusion, EDI-OCT imaging for SFCT measurement has recently emerged as a powerful adjunct to pathogenesis and prognosis and remains a research focus. However, a linear artifact is located stably at a depth of 485 *μ*m beneath the retinal pigment epithelium in 81.88% of subjects which might confound the measurement of SFCT. Images must be assessed with care and awareness of the existence of linear artifacts when determining the sclerochoroidal interface, especially when it is approximately 485 *μ*m.

## Methods

This study was approved by the Human Ethics Committee of Zhongshan ophthalmic center, and informed consent was obtained from all participants in accordance with the Declaration of Helsinki. All experiments were performed in accordance with relevant guidelines and regulations.

### Subjects

**Figure 3:**
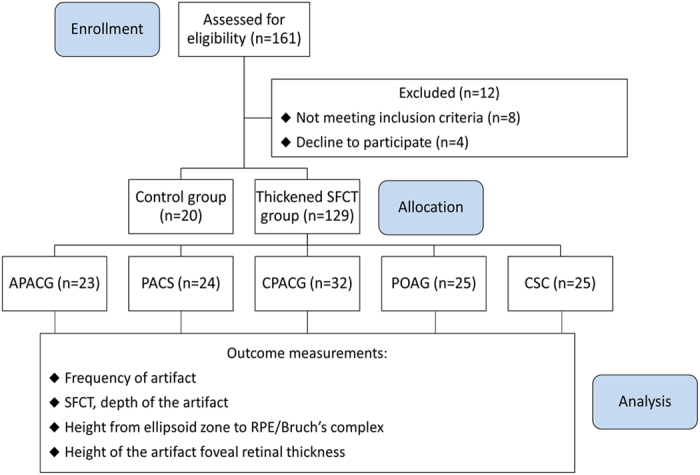
Summary and flow diagram of the study design (n: number of eyes). APACG: acute primary angle closure glaucoma; PACS: primary angle closure suspect; CPACG: chronic primary angle closure glaucoma; POAG: primary open angle glaucoma; CSC: central serous chorioretinopathy; SFCT: subfoveal choroidal thickness.

This was a prospective, consecutive, investigator-masked, nonrandomized, cohort study and was performed at a single center (Fig. 3). Twenty healthy subjects and 129 patients were enrolled in this study. There were76 men and 73 women. The mean age of the participants was 55.44 ± 13.46 years (range, 24–78 years), and the best-corrected visual acuity was 20/20 to hand motions. Eligibility criteria were: 1) normal healthy subjects and 2) patients with APACG, PACS, CPACG, POAG and CSC. Exclusion criteria were: 1) Significant corneal or media opacity; 2) Diabetes; 3) Uncontrolled hypertension (systolic > 150 mmHg and diastolic > 90 mmHg); 4) Amblyopia; 5) Neurologic or systemic disease that could compromise vision; 6) medications that are known to affect retinal structure; 7) Physical and/or mental impairment; or 8) Inability to sign a consent form. None of them had history of high myopia, ocular trauma, or ocular surgery.

### Measurement of linear artifact and subfoveal choroidal thickness

All participants were examined by two retinal specialists independently of each other. Two high-quality horizontal and vertical 9 *mm* EDI line scans across the fovea were obtained from each eye using the spectral-domain OCT device (wavelength: 870 nm; scan pattern: enhanced depth imaging; Spectralis; Heidelberg Engineering, Heidelberg, Germany). Eye motion artifacts were eliminated by a proprietary eye tracking device, and 100 frames of EDI OCT images were captured and automatically averaged to reduce speckle noise.

**Figure 4:**
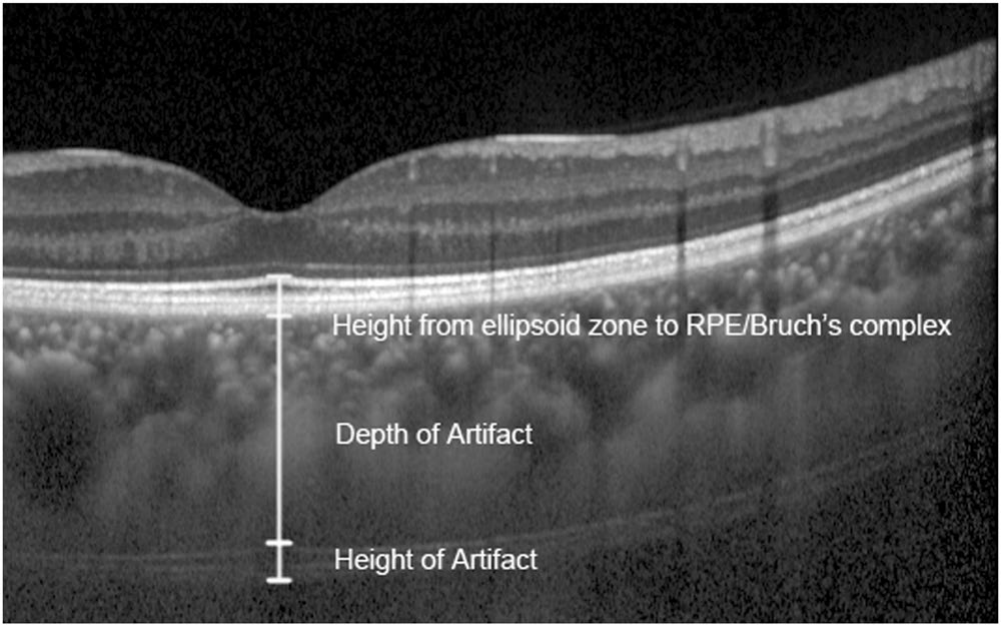
The linear artifact line measured by EDI-OCT. The height from the ellipsoid zone to RPE/Bruch’s complex and the depth and height of the linear artifact are demonstrated. RPE: retinal pigment epithelium.

The SFCT was measured using the manual calipers of the proprietary device, was taken at the fovea, and was defined as the distance between the outer part of the hyper-reflective line corresponding to the base of the RPE and the hypo-reflective line or margin corresponding to the sclerochoroidal interface^4^. The SFCT values of the horizontal and the vertical scans were averaged for analysis. The depth of subfoveal linear artifact was measured from the base of the RPE to the upper end of the three hyper-reflective bands similar to the ellipsoid zone, the interdigitation zone and the RPE/Bruch’s complex. As demonstrated in Fig. 4, the thickness of the linear artifact was defined as the distance between the upper and the lower end of the three hyper-reflective bands. Each OCT figure was reviewed by at least two of the readers. The SFCT and the depth and the thickness of the linear artifact were measured by at least two experienced readers independently and the values were averaged to get the reported results if the difference in the measurements of the readers was within 15% of the mean of the values. In a few cases, there was disagreement in measurement, and the determination was referred to Dr. Liu, the senior author.

### Statistical analysis

Statistical analysis was performed using absolute frequency (n) and relative frequency (%) for the qualitative variables and the mean ± standard deviation for quantitative variables. T-test was used for comparing the differences of SFCT between control subjects and thickened SFCT group. A p value less than 0.05 was considered statistically significant. All statistical tests were performed using SPSS Statistical Software, Release 20 (Chicago, IL, USA).

### Data availability

All data generated or analysed during this study are included in this published article.
